# Effect of Fe Content and Post-Heat Treatment on Mechanical and Corrosion Properties of Ti-5Mo-xFe (x = 2, 4 wt%) Alloys Fabricated by Hydrogenation–Dehydrogenation Process

**DOI:** 10.3390/ma19091813

**Published:** 2026-04-29

**Authors:** Jeong-Yeon Park, Min Kang, Ji-Hwan Park, Dong-Geun Lee

**Affiliations:** 1 Department of Materials Science and Metallurgical Engineering, Sunchon National University, 255, Jungang-ro, Suncheon 57922, Republic of Korea; 1230025@s.scnu.ac.kr; 2 Material Technical Innovation Group, 98, Nonhyeon-ro, Gangnam-gu, Seoul, Republic of Korea

**Keywords:** hydrogenation–dehydrogenation process, Ti-Mo-Fe alloy, pore fraction, corrosion resistance, mechanical properties

## Abstract

Cost-effective β-titanium alloys were developed via the hydrogenation–dehydrogenation (HDH) process using low-cost β-stabilizers Mo and Fe. Ti-5Mo-xFe (x = 2, 4 wt%) alloys were fabricated by powder metallurgy and subjected to six post-heat treatment conditions to reduce porosity and improve properties. The as-sintered alloys exhibited high porosity (15–20%), which adversely affected mechanical and corrosion performance. Heat treatment above the β-transus significantly reduced porosity, with Ti-5Mo-4Fe treated at 900 °C for 2 h showing the greatest reduction. Microstructures evolved from α + β lamellar Widmanstätten to equiaxed β with TiFe precipitates. Increased Fe content and heat-treatment temperature enhanced strength, while TiFe precipitates degraded corrosion resistance. Thus, optimized post-heat treatment improves strength and corrosion performance, although Fe content must be controlled.

## 1. Introduction

Titanium and its alloys are widely utilized in aerospace, biomedical, chemical engineering, and marine industries owing to their excellent mechanical properties, high corrosion resistance, and biocompatibility [1,2,3]. Among them, β-type Ti alloys, which possess a body-centered cubic (BCC) crystal structure, have gained particular attention for biomedical implants and structural applications due to their relatively low elastic modulus, high ductility, and superior strength [4,5]. However, β-type Ti alloys often contain expensive alloying elements such as Ta, Nb, Zr, and Hf, which substantially elevate production costs and limit their commercialization and large-scale manufacturing [6]. To address these challenges, the development of β-Ti alloys incorporating relatively low-cost alloying elements, along with the optimization of cost-effective manufacturing processes are essential.

In this study, Ti-Mo-Fe alloys were developed using molybdenum (Mo) and iron (Fe) as alloying elements to produce β-type Ti alloys with reduced material costs. Mo is a typical β-stabilizing element that enhances corrosion resistance through the formation of MoO_3_, while Fe increases the stability of the β phase and improves workability, high-temperature stability, and toughness [7]. The microstructure of Ti-Mo-Fe alloys is highly sensitive to heat treatment conditions, which considerably affect their mechanical properties and corrosion resistance.

The hydrogenation–dehydrogenation (HDH) process employed in this study produces high-purity titanium powder from low-cost titanium scrap, thereby enhancing cost competitiveness by recycling scrap materials [8,9]. Additionally, powder metallurgy (PM) processing techniques were employed to ensure the formability and processability of the materials. However, alloys fabricated via the PM route may exhibit poor mechanical properties owing to residual porosity. Therefore, the application of a suitable post-heat treatment process is essential to minimize the porosity, control the microstructure, and enhance mechanical and corrosion properties. In this study, Ti-5Mo-xFe (x = 2, 4 wt%) alloys were fabricated via the PM route using HDH-produced titanium powder. Particular emphasis was placed on controlling porosity through post-heat treatment conducted at temperatures above and below the β-transus. The effects of post-heat treatment conditions (temperature and holding time) on microstructural evolution, porosity reduction, and the resulting mechanical and corrosion properties were systematically investigated. Through this approach, the relationship between β-transus-controlled heat treatment, porosity evolution, and material performance was systematically elucidated.

## 2. Experimental

### 2.1. Alloy Fabrication and Selection of Post-Heat Treatment Conditions

**Figure 1:**
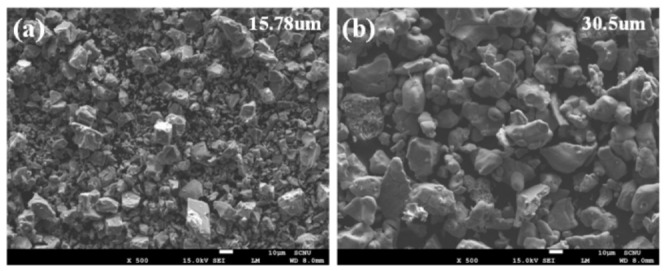
SEM images of (**a**) commercial titanium powder and (**b**) newly developed titanium powder.

The morphologies of the powders produced by HDH were examined using scanning electron microscopy (SEM; JSM-7001F, JEOL, Tokyo, Japan), as shown in Figure 1. The developed titanium powders had an average particle size of 30.5 μm, which is approximately twice that of the commercial titanium powders (15.78 μm). Metastable β-type Ti-5Mo-xFe (x = 2, 4 wt%) alloys were fabricated using the PM process, employing the developed titanium powder, molybdenum (Mo) powder with a particle size of 1–2 μm, and iron (Fe) powder with a particle size of 5–7 μm. The powder mixtures were compacted at a pressure of 280 kg/cm^2^ for 30 s and subsequently sintered at 1250 °C for 6 h, followed by furnace cooling, yielding alloy specimens with dimensions of 120 × 10 × 5 mm. In this study, porosity was controlled to enhance the strength, ductility, and toughness of alloys fabricated by PM and to mitigate the detrimental effects of residual pores on mechanical performance. The primary objective was to identify the optimal post-heat treatment conditions for maximizing the reliability and mechanical properties of the material. Each alloy exhibited a relatively high initial porosity, ranging from 15% to 20%, which was expected to negatively affect mechanical performance. Therefore, post-heat treatment was applied to reduce porosity. The β-transus temperatures of the Ti-5Mo-xFe (x = 2, 4 wt%) alloys were calculated using the empirical β-transus Equation (1), and the results guided the selection of appropriate post-heat treatment conditions [10].

T_β_ = 872 + 23.4[Al] + 32.1[Si] − 7.7[Mo] − 12.4[V] − 14.3[Cr] − 8.4[Fe] − 4.3[Zr] (1)

The calculated β-transus temperatures were approximately 816.7 °C for Ti-5Mo-2Fe and 799.9 °C for Ti-5Mo-4Fe. Based on these values, six different post-heat treatment conditions were applied: (#1) 750 °C/30 min, (#2) 750 °C/2 h, (#3) 830 °C/30 min, (#4) 830 °C/2 h, (#5) 900 °C/30 min, and (#6) 900 °C/2 h. After heat treatment, the porosity of each specimen was measured to evaluate the effects of the different conditions.

### 2.2. Microstructural Analysis

The microstructural evolution under different post-heat treatment conditions was analyzed using an optical microscope (OM; BX53M, Olympus, Tokyo, Japan) and a scanning electron microscope (SEM; JSM-7001F, JEOL, Japan). The experimental procedure was as follows. Ti-5Mo-xFe (x = 2, 4 wt%) alloy specimens were sectioned perpendicular to the longitudinal axis and hot mounted. The mounted samples were sequentially ground using abrasive papers of #220, #400, #800, #1200, and #2000 grit sizes, followed by polishing with 6 μm, 3 μm, 1 μm, and 0.04 μm colloidal silica suspension to achieve a mirror-like surface. Specimens were then etched using a solution of distilled water (H_2_O 100 mL), nitric acid (5 mL), and hydrofluoric acid (2 mL). Phase analysis for each post-heat treatment condition was performed using an X-ray diffraction (XRD; XRD-7000, Bruker D8, Billerica, MA, USA). XRD measurements were conducted over a 2θ range of 30° to 90°, with a step size of 0.02°. The phase volume fractions of α, α″, and β phases were calculated using the Equation (2), where V*_f_*_,*α*_, V*_f_*_,*α*″_, and V*_f_*_,*β*_ represent the volume fractions of the α, α″, and β phases, respectively, and A_α_, A_α″_, and A_β_ denote the total integrated areas of the respective phase peaks.

(2) $$V_{f,\;\alpha }=\frac{A_{\alpha }}{A_{\alpha }+A_{\alpha^{\prime \prime }}+A_{\beta }},\;V_{f,\;\alpha^{\prime \prime }}=\frac{A_{\alpha^{\prime \prime }}}{A_{\alpha }+A_{\alpha^{\prime \prime }}+A_{\beta }},\;V_{f,\;\beta \;}=\frac{A_{\beta }}{A_{\alpha }+A_{\alpha^{\prime \prime }}+A_{\beta }}$$

### 2.3. Evaluation of Mechanical Properties

To evaluate the mechanical properties under various post-heat treatment conditions, Vickers hardness measurements and room-temperature compression tests were performed. The Vickers hardness was assessed using a microhardness tester (HM-200, Mitutoyo, Tokyo, Japan) on the cross-sectional surface under a load of 1 kgf for 15 s. For each specimen, the hardness was measured at 20 points, and the average and standard deviation were calculated after excluding the highest and lowest values. Room-temperature compression tests were conducted using a universal materials testing machine (BESTUTM-10MD, Ssaul Bestech, Ansan-si, Republic of Korea) to evaluate and analyze the compressive properties. Compression specimens were fabricated by electrical discharge machining (EDM) with dimensions of 4 × 4 × 6 mm. The tests were performed at a strain rate of 10^−3^/s.

### 2.4. Evaluation of Electrochemical Corrosion Properties

Corrosion behavior under different post-heat treatment conditions was evaluated through potentiodynamic and potentiostatic polarization tests. The specimens were cold-mounted using copper tape, and the exposed surfaces were sequentially ground with abrasive papers ranging from #200 to #2000 grit. Polarization tests were conducted using a potentiostat (SP-150, Bio-Logic Science Instruments, Seyssinet-Pariset, France). A platinum coil was used as the counter electrode (CE), and an Ag/AgCl electrode (3 M HCl) was used as the reference electrode (RE). All electrochemical measurements were performed in a 0.9% NaCl solution.

## 3. Results and Discussion

### 3.1. Porosity Measurement

To evaluate the porosity of the Ti-5Mo-xFe (x = 2, 4 wt.%) alloys with respect to the Fe content and post-heat treatment conditions, density measurements were performed using the Archimedes method, and the corresponding porosity values were calculated. The results are summarized in Table 1. A comparison among the fabricated alloys revealed that the reduction in porosity ranged from a minimum of 1% to a maximum of 8%, depending on the alloy composition and post-heat treatment conditions. The Ti-5Mo-4Fe alloy exhibited a greater reduction in porosity than the Ti-5Mo-2Fe alloy, which is attributed to the addition of Fe as an alloying element. As a eutectoid-type β-stabilizing element with a relatively high diffusion coefficient in the titanium lattice, Fe enhances atomic mobility, promoting atomic rearrangement along pore walls. This leads to the transformation of irregular pores into low curvature, spheroidized pores. During high-temperature diffusion, Fe atoms can migrate into or near pores, resulting in pore filling via atomic penetration [11,12]. Due to the combined effects of Fe addition and post-heat treatment conducted above the β-transus temperature, the Ti-5Mo-4Fe alloy under condition #6 exhibited the greatest porosity reduction of approximately 8%.

### 3.2. Microstructural Observation

**Figure 2:**
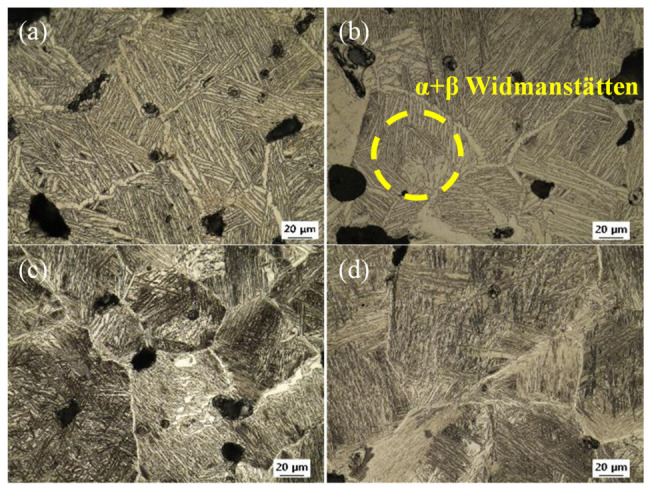
OM images of as-received Ti-5Mo-xFe (x = 2, 4 wt%) alloys: (**a**,**b**) Ti-5Mo-2Fe alloy and (**c**,**d**) Ti-5Mo-4Fe alloy.

**Figure 3:**
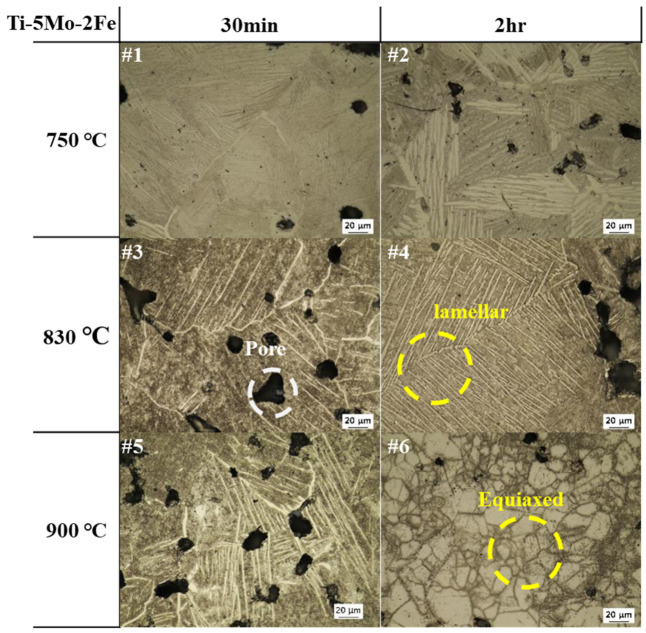
OM images of Ti-5Mo-2Fe alloy after post-heat treatment.

**Figure 4:**
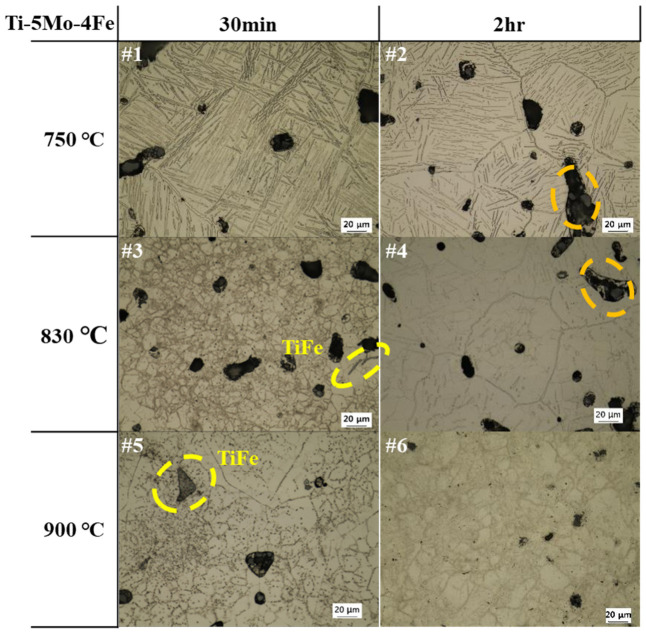
OM images of Ti-5Mo-4Fe alloy after post-heat treatment.

The microstructural changes in Ti-5Mo-xFe (x = 2, 4 wt%) alloys under various post-heat treatment conditions were observed using OM and SEM, as presented in Figure 2, Figure 3 and Figure 4. Microstructural observations revealed that the as-received specimens exhibited a Widmanstätten structure with an α + β lamellar configuration and relatively large pores measuring approximately 19.35 μm in size. In addition, with increasing Fe content, the spacing between the lamellar laths decreased from 3.51 ± 0.1 μm to 0.98 ± 0.3 μm due to the increased β-phase stability induced by Fe diffusion at elevated temperatures, promoting the formation of a more homogeneous β phase. This delayed or suppressed the nucleation of the α phase, leading to finer lamellar spacing. In Ti-5Mo-2Fe alloys under conditions #1–#5 and Ti-5Mo-4Fe alloys under conditions #1 and #2, a Widmanstätten structure with an α + β lamellar configuration was observed. A noticeable reduction in lamellar lath spacing was evident with increasing temperature and duration. In the Ti-5Mo-2Fe alloy under condition #6 and the Ti-5Mo-4Fe alloys under conditions #3–#6, equiaxed β-phase structures were observed. Additionally, TiFe precipitates were detected in Ti-5Mo-4Fe alloys treated under conditions #3–#6. Under certain conditions, an increase in the size of certain pores was observed, likely due to enhanced atomic diffusion that caused adjacent pores to coalesce. The pore surface, which is the interface between the solid and gas phases, having high surface energy due to broken atomic bonds, tends to evolve in a manner that minimizes the surface energy. Consequently, smaller, high curvature pores merge into fewer, larger pores. Although the size of the individual pores increased, the overall porosity decreased, simplifying the pore structure. This phenomenon reduces the total surface energy associated with the pores and contributes to the thermodynamic stabilization of the pore structure [13]. Additionally, partial pore filling induced by diffusion during the post-heat treatment is observed in Figure 3 (#4) and Figure 4 (#2, #4).

### 3.3. Mechanical Properties

**Figure 5:**
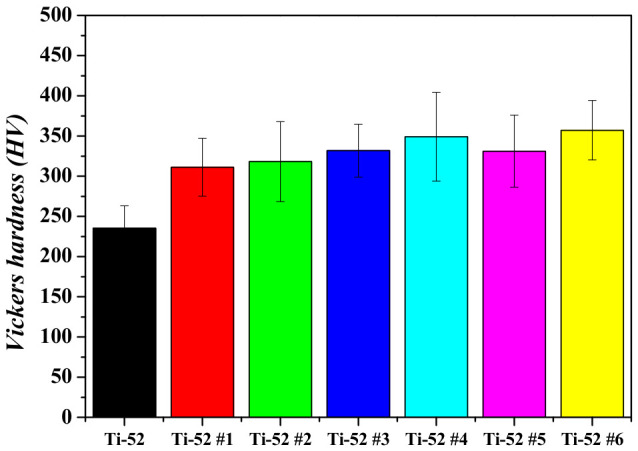
Vickers hardness of Ti-5Mo-2Fe alloy after post-heat treatment.

**Figure 6:**
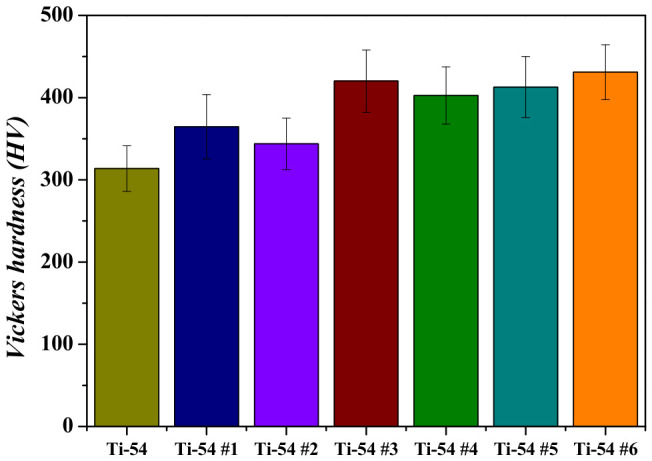
Vickers hardness of Ti-5Mo-4Fe alloy after post-heat treatment.

Vickers hardness and room-temperature compression tests were performed on Ti-5Mo-xFe (x = 2, 4 wt%) alloys. The results of the Vickers hardness tests are shown in Figure 5 and Figure 6 and Table 2. The as-received Ti-5Mo-4Fe alloy exhibited a hardness approximately 78.4 HV higher than that of the Ti-5Mo-2Fe alloy. This increase is attributed to the narrower α lamellar lath spacing and the solid solution strengthening effect in the Ti-5Mo-4Fe alloy [14]. The lamellar interfaces between crystallographic orientations and α/β phases act as barriers to dislocation motion due to differences in interfacial energy and elastic modulus. As the spacing becomes narrower, the boundary area per unit volume increases, leading to an increase in the hardness [15]. Among the heat-treated alloys, the Ti-5Mo-4Fe specimen under condition #6, which exhibited the greatest reduction in porosity, showed the highest hardness value of 431 HV.

The pore size of the Ti-5Mo-4Fe alloy under condition #6 was measured to be approximately 7.2 μm, the smallest among all conditions, which is considered to have contributed to the increased hardness. Additionally, across the entire range of post-heat treatment conditions, the Ti-5Mo-4Fe alloy exhibited superior hardness compared to the Ti-5Mo-2Fe alloy, which is attributed to increased Fe content.

**Figure 7:**
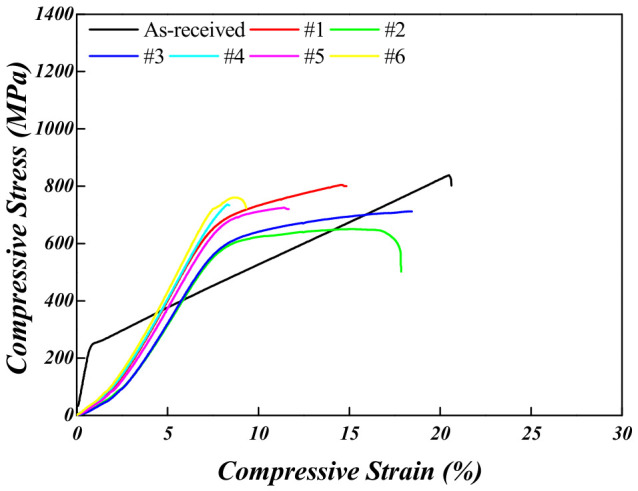
Room-temperature compressive stress–strain curves of Ti-5Mo-2Fe alloy after post-heat treatment.

**Figure 8:**
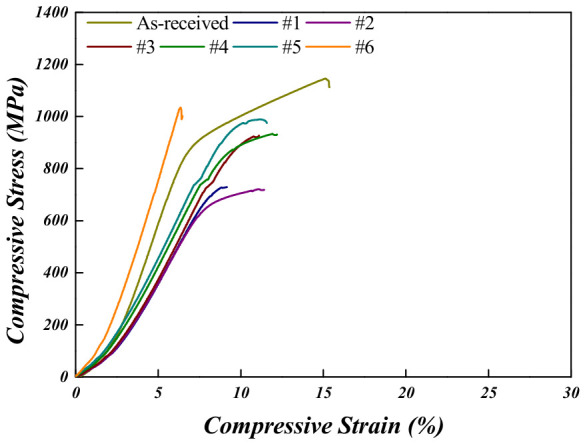
Room-temperature compressive stress–strain curves of Ti-5Mo-4Fe alloy after post-heat treatment.

Room-temperature compression tests were conducted to further evaluate the mechanical properties in correlation with the Vickers hardness results. The results of these tests are presented in Figure 7 and Figure 8 and are summarized in Table 3 and Table 4. As a result, the Ti-5Mo-2Fe alloy in the as-received condition exhibited a compressive yield strength of 262 ± 21.3 MPa, maximum compressive strength of 836 ± 11.5 MPa, and compressive strain of 20.4 ± 1.2%. Although the as-received condition was expected to exhibit poor mechanical properties owing to its high porosity of approximately 20%, the room-temperature compression test revealed that it demonstrated a higher compressive strain than the samples that underwent post-heat treatment and exhibited lower porosity. This behavior is attributed to the presence of internal pores, which appear to increase the apparent ductility. During compressive loading, the gradual collapse and densification of the pores allowed for greater plastic deformation prior to fracture. In contrast to tensile loading, pores act as stress concentrators, leading to reduced ductility. Under compression, pores function as buffer zones that provide deformation allowance, thereby contributing to enhanced compressive strain.

Under post-heat treatment conditions, higher temperatures and longer holding times led to an increase in the maximum compressive strength, accompanied by a reduction in compressive strain. The Ti-5Mo-xFe (x = 2, 4 wt%) alloys are classified as metastable β-type titanium alloys, in which the β phase, having a body-centered cubic (BCC) structure, possesses more available slip systems compared to the α phase. As a result, a higher β-phase fraction contributes to increased compressive strength [16,17]. Additionally, the microstructure of the Ti-5Mo-2Fe alloy under condition #4, which was heat-treated below the β-transus temperature, exhibited a fine α + β lamellar structure with extremely narrow α lath spacing. In this structure, dislocations frequently interact with the α/β interfaces, which act as stress concentration sites and facilitate crack propagation along specific directions. Consequently, premature fractures were observed prior to the yield point [18].

**Figure 9:**
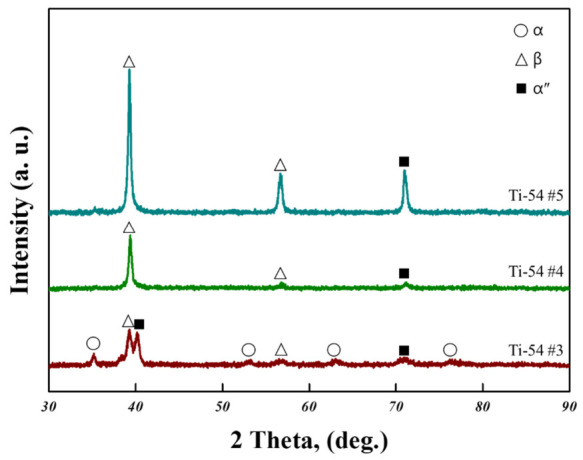
X-ray diffraction patterns of Ti-5Mo-4Fe alloy after post-heat treatment.

In the as-received condition, the Ti-5Mo-4Fe alloy exhibited a compressive yield strength of 932 ± 9.0 MPa, a maximum compressive strength of 1145 ± 19.5 MPa, and a compressive strain of 15.2 ± 2.4%. When comparing the Ti-5Mo-4Fe and Ti-5Mo-2Fe alloys under all post-heat treatment conditions, the Ti-5Mo-4Fe alloy consistently exhibited superior compressive properties. This is attributed to the effect of Fe addition, in which Fe atoms are dissolved into the Ti lattice, causing lattice distortion and hindering dislocation movement, thereby making plastic deformation more difficult. In other words, Fe increases lattice friction, which contributes to strengthening the alloy [11,19]. For Ti-5Mo-4Fe alloys under conditions #3, #4, and #5, the compression test results revealed double yielding. Double yielding refers to the phenomenon observed in the stress–strain curve of a material where two distinct yield points appear. After the initial yielding beyond the elastic region, the stress increased again, followed by a second nonlinear deformation stage. This behavior is generally associated with the presence of composite phase microstructures or microstructural phenomena such as stress-induced martensitic (SIM) transformation [20]. The XRD patterns used to investigate the phases that precipitated after compression under each condition are shown in Figure 9. The SIM transformation is known to occur when the unstable β phase undergoes deformation under external loading, resulting in a phase transformation into the α″ phase and the formation of an α + β + α″ microstructure [21]. In all conditions where double yielding was observed, α″ phase peaks were detected, indicating that the SIM transformation from β to α″ was induced by the stress generated during compression. Additionally, Table 5 presents the phase fractions calculated from the XRD patterns, showing that approximately 20% of the α″ phase was present under all conditions. These findings support the conclusion that the observed double-yielding behavior was induced by SIM transformation. Specifically, the first yield point corresponds to plastic deformation within the β phase, while the second yield point is attributed to additional deformation associated with the formation of the α″ phase from the β matrix. However, the α″ phase fractions in the Ti-5Mo-4Fe alloys under conditions #4 and #5 (V*_f_*_,*α*″_ = 10.3% and 20.8%) were relatively lower than that of condition #3 (V*_f_*_,*α*″_ = 34.7%). This reduction in α″ fraction is attributed to the increased β-phase stability induced by the higher temperature and prolonged duration of the post-heat treatment. As β stability increases, the chemical driving force for the α″ transformation decreases, thereby suppressing the formation of the α″ phase and resulting in a lower volume fraction [22,23].

### 3.4. Electrochemical Corrosion Properties

**Figure 10:**
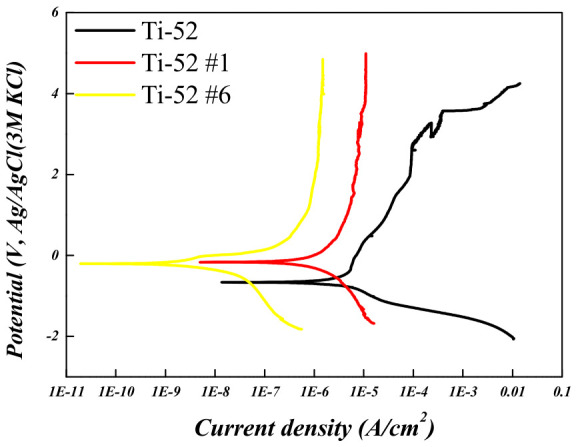
Potentiodynamic polarization curves of Ti-5Mo-2Fe alloy after post-heat treatment.

**Figure 11:**
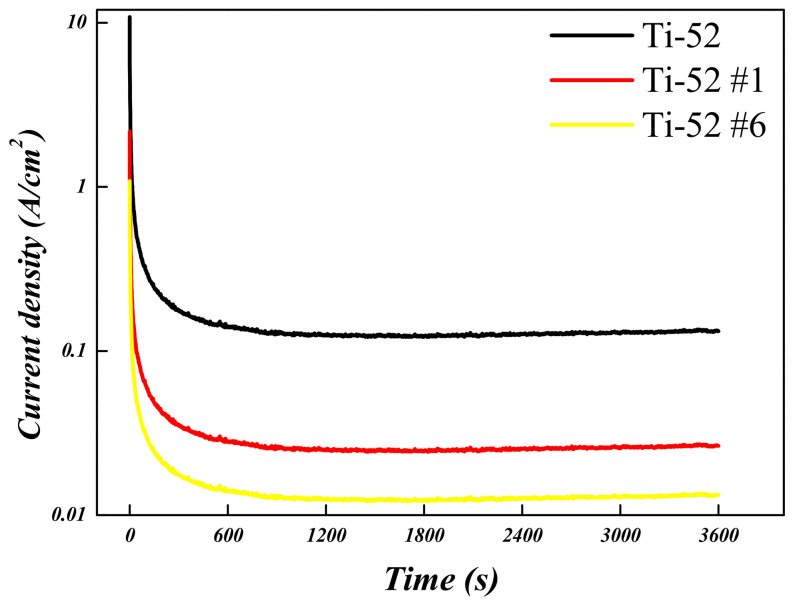
Potentiostatic polarization curves of Ti-5Mo-2Fe alloy after post-heat treatment.

**Figure 12:**
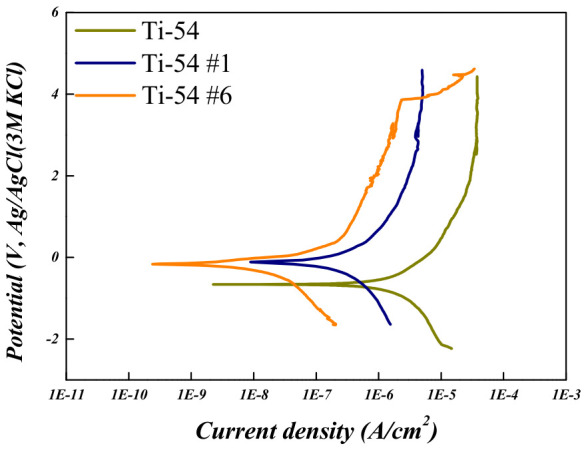
Potentiodynamic polarization curves of Ti-5Mo-4Fe alloy after post-heat treatment.

**Figure 13:**
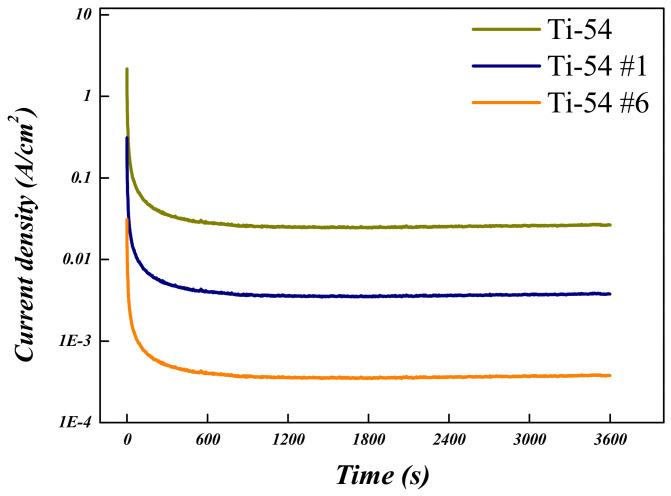
Potentiostatic polarization curves of Ti-5Mo-4Fe alloy after post-heat treatment.

**Figure 14:**
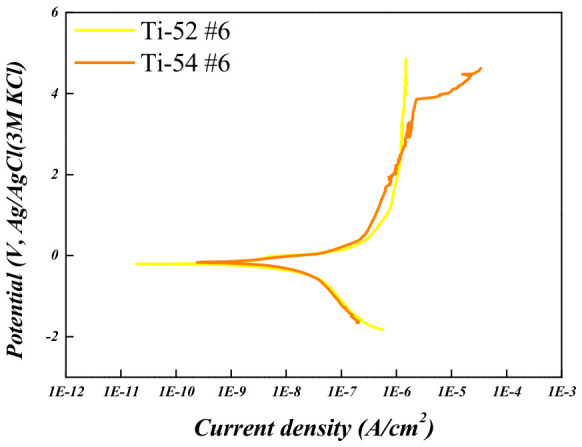
Potentiodynamic polarization curves of Ti-5Mo-xFe (x = 2, 4 wt%) alloys under condition #6.

To evaluate the corrosion behavior of the Ti-5Mo-xFe (x = 2, 4 wt%) alloys, potentiodynamic and potentiostatic polarization tests were conducted on the as-received specimens as well as on those subjected to post-heat treatment under conditions #1 and #6, which exhibited the smallest and largest reductions in porosity, respectively. The results are presented in Figure 10, Figure 11, Figure 12 and Figure 13 and in Table 6 and Table 7. The corrosion resistance of the Ti-5Mo-xFe (x = 2, 4 wt%) alloys improved as the porosity decreased, with each alloy exhibiting enhanced corrosion properties under conditions of reduced porosity. The interior of the pores provides an environment with a limited oxygen supply, leading to the formation of electrochemical conditions distinct from those at the surface. Consequently, the interior of the pores acted as a cathode, whereas the exterior functioned as an anode, promoting the occurrence of localized corrosion. Titanium alloys generally exhibit excellent corrosion resistance owing to the formation of a stable passive film [24]. However, near the pores, the passive film may not form uniformly and may be disrupted. This discontinuity in the passive film can lead to localized corrosion and mechanical degradation in the affected regions [25]. In addition, typical pores act as stress concentration sites within the material and can initiate stress corrosion cracking. According to the potentiodynamic polarization results, both the Ti-5Mo-2Fe as-received condition and the Ti-5Mo-4Fe alloy under condition #6 exhibited a sharp increase in current density beyond a certain potential range during oxidation. This behavior is indicative of the onset of localized corrosion. However, no repassivation or plateau behavior was observed beyond the critical potential range, suggesting that the previously mentioned pores contributed to nonuniform passive film formation and localized damage. These factors are believed to sustain the unstable oxidation behavior. Subsequently, the experimental results of the two conditions that exhibited the greatest reduction in porosity for each alloy, namely Ti-5Mo-2Fe condition #6 and Ti-5Mo-4Fe condition #6, were compared and evaluated, as shown in Figure 14. Although the Ti-5Mo-4Fe alloy under condition #6 exhibited the lowest porosity of approximately 7%, its corrosion behavior was nearly equivalent to that of the Ti-5Mo-2Fe alloy under the same conditions. This can be attributed to the increased Fe content, which facilitated the precipitation of intermetallic compounds such as TiFe. These precipitates are known to impair the formation of a stable passive film and promote galvanic corrosion, ultimately leading to a deterioration in the corrosion resistance [26,27].

## 4. Conclusions

In this study, Ti-5Mo-xFe (x = 2, 4 wt%) powder alloys were fabricated using the HDH process and relatively low-cost β-stabilizing elements, Mo and Fe, to enhance cost competitiveness. The pores generated during this process led to degradation of material properties; therefore, post-heat treatment was applied to control the porosity. The effects of the post-heat treatment conditions (temperature and duration) on the porosity, microstructure, mechanical properties, and electrochemical corrosion properties were analyzed:

1. The post-heat treatment process was generally effective in reducing porosity, with the greatest reduction observed in the Ti-5Mo-4Fe alloy under condition #6, which involved treatment above the β-transus temperature for an extended duration, resulting in an 8% decrease in porosity.

2. Under post-heat treatment conditions below the β-transus temperature, an α + β lamellar structure was observed, with the lamellar lath spacing decreasing as the temperature and holding time increased. In contrast, treatments conducted above the β-transus temperature resulted in the formation of an equiaxed β-phase microstructure, accompanied by the precipitation of TiFe intermetallic compounds.

3. Both the hardness and compressive strength were enhanced with increasing Fe content and high-temperature post-heat treatment conditions. The Ti-5Mo-4Fe alloy exhibited the highest hardness (431 HV) and compressive strength (1145 ± 19.5 MPa), which could be attributed to the narrow lamellar lath spacing, reduced porosity, and strengthening effect of the precipitates.

4. Under conditions in which porosity was reduced through post-heat treatment, an overall improvement in corrosion resistance was observed. However, the Ti-5Mo-4Fe alloy under condition #6, which contained a higher Fe content, exhibited a corrosion behavior similar to that of the Ti-5Mo-2Fe alloy under the same conditions. This was attributed to the non-uniform passive film formation and micro-galvanic effects caused by the TiFe precipitates, indicating that excessive Fe content can have a detrimental effect on corrosion resistance.

## Figures and Tables

**Table 1:** Measured porosity fractions of Ti-5Mo-xFe (x = 2, 4 wt%) alloys.

		Before Post-Heat Treatment	After Post-Heat Treatment
	ID	Pore Fraction	Pore Fraction
**Ti-5Mo-2Fe**	**Ti-52**	20%	20%
	**Ti-52 #1**	17%	16%
	**Ti-52 #2**	17%	16%
	**Ti-52 #3**	17%	15%
	**Ti-52 #4**	17%	16%
	**Ti-52 #5**	16%	13%
	**Ti-52 #6**	18%	13%
**Ti-5Mo-4Fe**	**Ti-54**	17%	17%
	**Ti-54 #1**	15%	13%
	**Ti-54 #2**	14%	12%
	**Ti-54 #3**	15%	12%
	**Ti-54 #4**	15%	11%
	**Ti-54 #5**	18%	12%
	**Ti-54 #6**	15%	7%

**Table 2:** Vickers hardness of Ti-5Mo-xFe (x = 2, 4 wt%) alloys after post-heat treatment.

	ID	Vickers Hardness (HV)	Standard Deviation (HV)
**Ti-5Mo-2Fe**	**Ti-52**	235	±27.9
	**Ti-52 #1**	311	±35.9
	**Ti-52 #2**	318	±49.7
	**Ti-52 #3**	332	±33.0
	**Ti-52 #4**	349	±55.3
	**Ti-52 #5**	331	±44.8
	**Ti-52 #6**	357	±37.0
**Ti-5Mo-4Fe**	**Ti-54**	314	±27.9
	**Ti-54 #1**	365	±39.1
	**Ti-54 #2**	344	±31.4
	**Ti-54 #3**	420	±37.9
	**Ti-54 #4**	403	±34.8
	**Ti-54 #5**	413	±37.2
	**Ti-54 #6**	431	±33.4

**Table 3:** Room-temperature compression test data of Ti-5Mo-2Fe alloy.

Ti-5Mo-2Fe	Compressive Yield Strength (MPa)	Maximum Compressive Strength (MPa)	Compressive Strain (%)
**As-received**	262 ± 21.3	836 ± 11.5	20.4 ± 1.2
**#1**	686 ± 10.5	805 ± 25.8	14.6 ± 2.5
**#2**	598 ± 24.2	645 ± 10.8	16.7 ± 2.1
**#3**	644 ± 10.5	712 ± 27.5	18.5 ± 1.8
**#4**	-	736 ± 10.0	8.3 ± 3.4
**#5**	686 ± 20.2	725 ± 22.5	12.1 ± 1.2
**#6**	729 ± 14.1	760 ± 23.0	8.7 ± 1.1

**Table 4:** Room-temperature compression test data of Ti-5Mo-4Fe alloy.

Ti-5Mo-4Fe	Compressive Yield Strength (MPa)	Maximum Compressive Strength (MPa)	Compressive Strain (%)
**As-received**	932 ± 9.0	1145 ± 19.5	15.2 ± 2.4
**#1**	-	1032 ± 9.2	6.3 ± 1.1
**#2**	699 ± 20.2	720 ± 10.2	10.9 ± 2.0
**#3**	910 ± 11.1	919 ± 5.0	11.1 ± 1.0
**#4**	978 ± 14.1	988 ± 10.8	11.0 ± 1.4
**#5**	920 ± 13.4	932 ± 29.1	11.8 ± 2.5
**#6**	-	1027 ± 30.5	8.8 ± 1.3

**Table 5:** Volume fraction (%) of α, β, and α″ phase in Ti-5Mo-4Fe alloy.

	V*_f_*_,*α*_ (%)	V*_f_*_,*β*_ (%)	V*_f_*_,*α*″_ (%)
**Ti-54 #3**	22.3	43.0	34.7
**Ti-54 #4**	-	89.7	10.3
**Ti-54 #5**	-	79.2	20.8

**Table 6:** Electrochemical corrosion test data of Ti-5Mo-2Fe alloy.

	E_corr_ (V)	I_corr_ (A/cm^2^)	Corrosion Rate (mmy^−1^)
**Ti-52**	−0.664	11.1 × 10^−7^	14.23 × 10^−11^
**Ti-52 #1**	−0.166	5.62 × 10^−7^	6.94 × 10^−11^
**Ti-52 #6**	−0.195	4.54 × 10^−9^	5.64 × 10^−13^

**Table 7:** Electrochemical corrosion test data of Ti-5Mo-4Fe alloy.

	E_corr_ (V)	I_corr_ (A/cm^2^)	Corrosion Rate (mmy^−1^)
**Ti-54**	−0.669	24.1 × 10^−8^	291 × 10^−12^
**Ti-54 #1**	−0.108	7.42 × 10^−8^	8.72 × 10^−12^
**Ti-54 #6**	−0.153	3.18 × 10^−9^	3.77 × 10^−13^

## Data Availability

The raw data supporting the conclusions of this article will be made available by the authors on request.
